# Quality control methods for *Aedes albopictus* sterile male production

**DOI:** 10.1371/journal.pntd.0005881

**Published:** 2017-09-11

**Authors:** Fabrizio Balestrino, Arianna Puggioli, Marco Carrieri, Jérémy Bouyer, Romeo Bellini

**Affiliations:** 1 Insect Pest Control Laboratory, NAFA Joint FAO/IAEA Division of Nuclear Techniques in Food and Agriculture, FAO/IAEA Agriculture and Biotechnology Laboratories, Seibersdorf, Austria; 2 Medical and Veterinary Entomology Department, Centro Agricoltura Ambiente CAA "G. Nicoli”, Crevalcore, Italy; Liverpool School of Tropical Medicine, UNITED KINGDOM

## Abstract

The capacity of the released sterile males to survive, disperse, compete with wild males and inseminate wild females is an essential prerequisite to be evaluated in any area-wide integrated pest management (AW-IPM) programs including a sterile insect release method. Adequate quality control tests supported by standardized procedures need to be developed to measure these parameters and to identify and correct potential inappropriate rearing or handling methods affecting the overall male quality. In this study, we report results on the creation and validation of the first quality control devices designed to infer the survival and mating capacity of radio-sterilized *Aedes albopictus* males through the observation of their flight capacity under restricted conditions (flight organ device) and after stress treatment (aspirator device). Results obtained consistently indicate comparable flight capacity and quality parameters between untreated and 35 Gy irradiated males while a negative impact was observed with higher radiation doses at all observation time performed. The male flight capacity registered with the proposed quality control devices can be successfully employed, with different predictive capacities and response time, to infer the adult male quality. These simple and cost-effective tools provide a valuable method to detect and amend potentially sub-standard procedures in the sterile male production line and hence contribute to maintaining optimal quality and field performance of the mosquitoes being released.

## Author summary

The absence of effective therapeutics and vaccines for emerging and re-emerging vector-borne diseases and the limited effectiveness of conventional vector control methods renovated the interest for the implementation and integration of the SIT and genetic control methods for successful area-wide integrated mosquito management strategies. These methods involve the release of mass reared insects genetically sterile, incompatible or resistant to pathogen amplification and transmission which need to effectively introduce in the wild target population adequate sterility or the desired genetically selected traits. The availability of easy and standardized quality control tests could facilitate the adoption of international procedures for mosquito strains evaluation and comparison and to assess strain suitability for effective mosquito genetic control applications.

## Introduction

The sterile insect technique (SIT) is a biological pest control method based on area-wide inundative releases of sterile insects to reduce the reproduction in a field population of the same species [1]. The SIT has been employed all around the world as part of area-wide integrated pest management programs (AW-IPM) over the past 60 years to contain, reduce, eliminate or prevent the establishment of insect pests of agronomic, veterinary and medical importance [2]. Despite some initial successful applications [3], the development of this promising pest control method against mosquitoes was mainly abandoned.

The absence of effective therapeutics and vaccines for emerging and reemerging vector-borne diseases and the limited effectiveness of conventional vector control methods renovated the interest for the implementation and integration of the SIT and other genetic-based control methods in area-wide integrated mosquito management strategies.

The creation of effective and stable sexing mechanisms and the development of effective mosquito mass production and release methods are some of the principal aspects to be developed for the practical integration of these technologies in mosquito control programs [4]. Beside the fundamental need to produce large quantities of males, it is essential that the released sterile males have the capacity to survive, actively disperse and compete for mating in the field. While the quantity can be easily measured, the quality of the insects produced is more difficult to estimate. Quality can be defined as the compliance between defined and detected characteristics of a product as determined by performing tests. We can similarly identify the quality of an insect only if a dedicated test measurement ascertains that its selected desired characteristics lie within a given tolerance range [5, 6].

The importance of quality control procedure for the successful application of any SIT program is today widely recognized. Standardized quality control tests and procedure are today routinely and strictly observed in all successful operational SIT programs to ensure adequate quality of the sterile mass produced insect and to facilitate results comparability. The qualitative standards of the insect produced have to be assured through a system of bioassays of quality parameters in order to routinely assure adequate performance and competitiveness after release. Adult emergence rate, flight and dispersal capacity, longevity, location of mating arenas, courtship, mating and sperm transfer are the most important parameters to be monitored and preserved during any campaign involving the release of sterile mass reared insects. The identification of deficit in these parameters may have a detrimental effect on the sterile insect release program effectiveness and require the adoption of corrective actions on the production line in order to restore adequate standard biological traits in the insect to be released [4, 5, 7–9].

The absence of standardized and mature technologies and procedures for the mass rearing of mosquito vector species delays the development of quality control methods for the evaluation of the final sterile male performance. The present quality control procedures for sterile mosquito males are therefore based on long and laborious laboratory, semi-field and field tests for the estimation of male flight potential, survival, dispersal capacity, mating ability and competitiveness [10–13]. Rapid and inexpensive laboratory tests capable of predicting and comparing mosquito male performance and to assess strain suitability for effective mosquito genetic control applications are therefore needed [5].

Flight capacity of insects is one of the most direct and reliable indicator of insect quality and the ability of an insect to fly out from simple flight tube devices is a standard quality control procedure regularly employed in the most advanced operational SIT programs [8, 14–16].

The possibility to evaluate, measure and compare the quality of mosquitoes through the analysis of their flight capacity was the main aim of this study. We report results on the creation and validation of the first quality control devices designed to infer the sterile *Aedes albopictus* male mosquito quality through the observation of their flight capacity under restricted conditions and after stress treatments and compare their results to reference tests of survival and mating capacity. Standardized quality control tests could facilitate the creation of international procedures for mosquito strain evaluation and comparison. These rapid and cost-saving tools are essential instruments to identify and correct inappropriate sterile insect rearing procedures and to evaluate single and cumulative stress along the male production line capable of affecting the final male quality and field performance.

## Methods

### Ethics statement

Research carried out on invertebrates such as mosquitoes do not require a specific permit according to the directive 2010/63/EU of the European Parliament and of the Council on the protection of animals used for scientific purposes. The experiments were performed inside the Biosafety Level 3 laboratory—BL3 (Ministry of Health, Italian Ministerial Legislative Decree, 626/94, Annex XII) of the Medical and Veterinary Entomology Department, Centro Agricoltura Ambiente “G. Nicoli”- IAEA collaborating Centre in Crevalcore, Italy, in compliance with to the Standard Operating Procedure in force in a mosquito laboratory. The blood used for routine blood-feeding was collected in Camposanto, Italy during routine slaughtering of pigs in a national authorized abattoir (Az. Agr. All. Rubizzani CE IT N2L7D) at the highest possible standards strictly following EU laws and regulations.

### Mosquito stocks and rearing method

The *Aedes albopictus* strain employed in this study was obtained from eggs collected in the field in urban areas of Rimini, Emilia-Romagna region, northern Italy (Rimini strain) and maintained for 60 generations under laboratory conditions (28 ± 1°C, 80 ± 5% RH, 14:10 h L:D photoperiod). Larvae obtained after standardized hatching procedures [17] were reared at a fixed larval density (2 larvae/ml) and fed with IAEA-BY liquid diet (5.0% w/v) at a mean daily dose of 0.5 mg/larvae [18] for the first four days of development. Pupae were harvested on the sixth day from larval introduction and males were separated using a 1400-micron sieve with 99.0% accuracy [19, 20]. Part of the selected larger pupae were collected and sexed under a stereomicroscope in order to obtain adequate number of females. Females were left to emerge inside a dedicated cage provided with daily access to 10% sucrose solution in order to preserve unmated status. All of the separated male pupae were aged at least 24 h before being subjected to the different irradiation treatments. All tests described were performed inside a climate-controlled chamber with temperature, humidity and lighting conditions as reported above.

### Irradiation procedure

Irradiation treatments were performed at the Medical Physics Department of St. Anna Hospital (Ferrara, Italy) using a gamma irradiator (IBL 437C, CIS Bio International, Bagnols sur Ceze, France; 65.564 TBq 1772 Ci ± 10% Cs-137 linear source) at a dose rate of 2.2 Gy/min (± 3.5%). At the irradiation facility male pupae were transferred to Petri dishes (12 cm diameter) with a minimum amount of water and placed in the center of the irradiation chamber to minimize dose variation. Calibration of the radiation source met the requirements of French and international norms: NF M 61002, ISO 1677 ISO 2919, NF ISO 9978, ANS N542. Time of irradiation for the different doses was calculated based on the decay table of the isotopic source routinely corrected.

### Flight organ device

**Fig 1:**
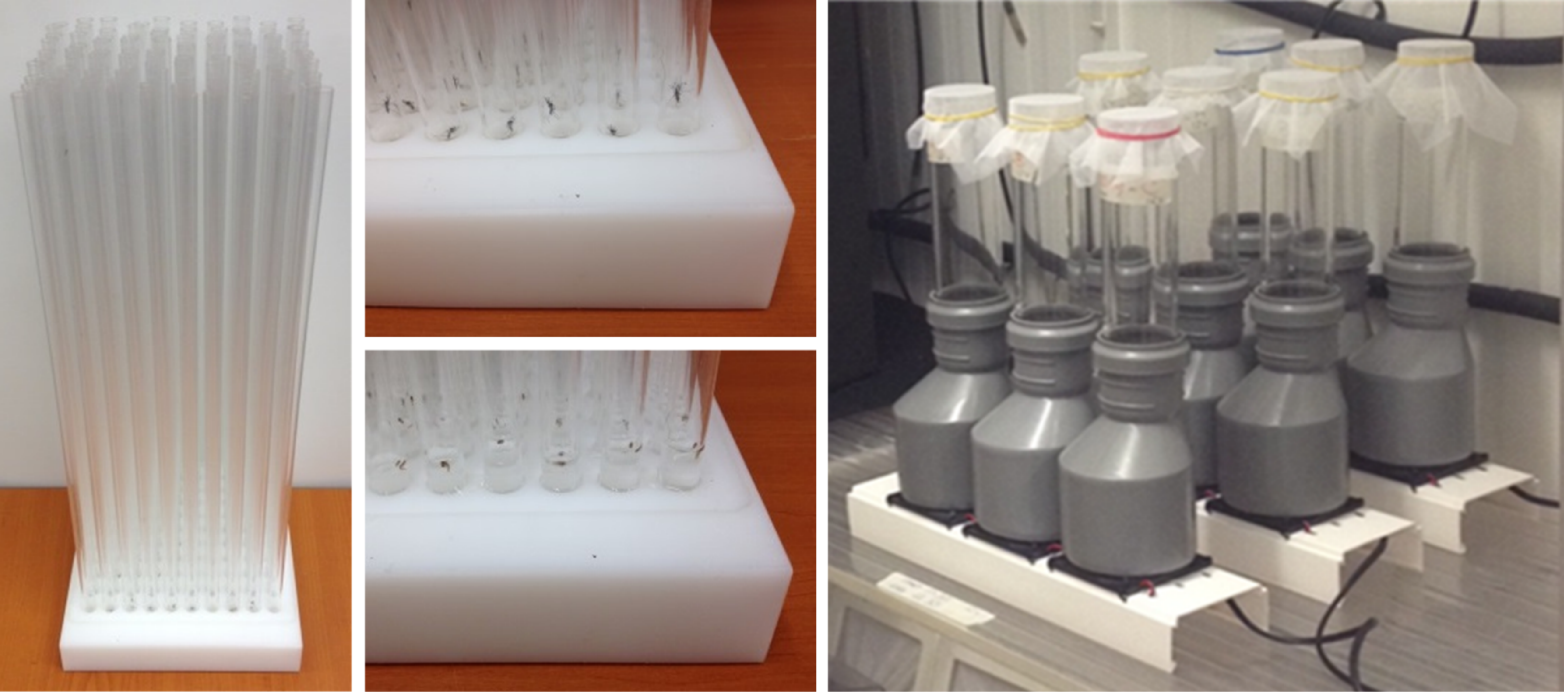
The flight organ (left and center) and the aspirator (right) employed as *Aedes albopictus* quality control devices.

A quality control device has been developed in order to evaluate the flight ability of adult male mosquitoes. This device (hereafter called flight organ) is formed by a plastic (Polytetrafluoroethylene, PTFE) base plate (170 x 170 x 30 mm) in which a group of 100 wells have been drilled (Fig 1, left and center). The wells (diameter 10.1 mm, depth 20 mm) can be filled with water in order to host a single pupa per well. After placing a pupa in each well, extruded transparent acrylic plastic (Polymethyl methacrylate, PMMA) tubes (outside diameter 10 mm, inside diameter 8 mm) can be introduced inside the wells without damaging the pupae. After emergence the reduced space and the low friction inner surface of the tubes compel the adults to fly out vertically without crawling. The rate and the time for male adult emergence, and escape from the tubes, can therefore be determined by routinely checking the presence of the pupae or adults inside the tubes. The flight organ device is conceived to be operated inside a clear acrylic plastic containment box in order to confine, quantify and sex the adult escaped from the tubes.

### Aspirator device

An additional quality control device was also created to conduct a rapid stress tolerance test for adult male mosquito. The stress test was introduced to highlight possible biological stress conditions making them more easily and rapidly detectable and measurable. This device consists of an aspirator fan (8.0 cm diameter, Jamicon—Model JF0825S1H-S, 12V DC, 0.19A, Kaimei Electronic Corp, Taiwan) mounted inside a plastic adaptor tube (Level Invert Reducer 110/50mm) and connected to a removable vertical suction tube (transparent acrylic plastic, 20 cm length, 5 cm diameter) (Fig 1, right). The suction tube is closed at the bottom with a fix plastic net (mesh size 1 mm) while the top opening can be plugged with a netted lid to collect and retain adult mosquitoes until the start of the test. When activated, the aspirator holds the mosquitoes against the suction tube bottom net inducing a stress detectable by their capacity to fly out of the suction tube at the end of the aspiration session. The stress induced on the insect can be regulated either by varying the aspiration time or modifying the aspiration power by adjusting the voltage supplied to the fan (Adjustable Voltage Regulator and AC/DC converter). Similarly to the flight organ the aspirator device need to be operated inside a containment box.

### Experiment 1: Flight organ validation tests

The flight organ validation tests aimed to evaluate the effect of forced restraint of pupae inside the flight organ on adult emergence rate and to establish a relationship between the height of the tubes and the time and rate of adult male escape. Male pupae used in this test were not subjected to irradiation procedures. All tests were performed inside a climate-controlled chamber with temperature, humidity and lighting conditions as above.

Tests were performed using transparent acrylic tubes of nine different heights equal to 5, 10, 15, 20, 25, 30, 35, 40 and 45 cm respectively. Ten tubes for each of these heights were created and used for each of the five replicates performed. Ninety wells per base plate had tubes of different heights inserted while the remaining 10 wells were left without tubes in order to evaluate the adult emergence rate of the pupae without the constriction of the tubes.

An additional 200 male pupae from the same cohort were processed and sexed as the one tested inside the flight organs and divided equally between 10 Petri dishes (diameter 90 mm) containing 35 ml of water as a control not affected by space limitation and isolation of the pupae.

Adult emergence rate was calculated as the ratio between the number of adult emerged and the initial number of pupae in the different settings. The adult emergence in the different flight organs tubes was recorded every two hours from pupal introduction in order to calculate adult escape time. The adult escape rate in the flight organs was calculated as the ratio between the number of adult escaped from the tubes and the total number of adult successfully emerged inside the flight organ. The number of dead pupae and the number of dead or trapped adults inside the tubes at the different height were recorded at the end of each trial.

### Experiment 2: Effect of radiation, tube height and time on adult male flight ability

Preliminary tests were conducted to evaluate the effect of different pupal radiation doses (0, 35 and 80 Gy) on adult male escape rate from tubes of 20, 40 and 80 cm of height at 48 h after the introduction of pupae. Three replicates per dose were performed for each different height investigated.

Based on the results obtained in the preliminary tests a second trial was performed to investigate the effect of different pupal radiation doses (0, 35, 60 and 80 Gy) on adult male escape rate from tubes of 40 and 80 cm height at 48, 72 and 120 h after the introduction of pupae. At least three replicates per dose were performed for each treatment.

All flight organs employed in these trials were fully loaded with pupae (100 pupae) and were positioned inside top opened black plastic box of the same height as the tubes to stimulate the positive phototactic response of the emerging adults in low light conditions.

### Experiment 3: Effect of radiation on adult male stress resistance

The capacity of adult males to recover from stress conditions was investigated on samples of 40 adult males aged 24 h after treatments with different radiation doses (0, 35, 60 and 80 Gy). Males were exposed for 1, 2 or 3 hours of aspiration at the maximum fan capacity (12V DC) of the aspirator device. Under this condition the mean (± SD) air flow speed measured at the suction tube net level (Testo 405-V1 Thermo-Anemometer, Sparta, NJ) was 2.51 (± 0.12) m/s for the nine aspirator devices built and employed in these tests. The adult escape rate from the suction tube after each aspiration session was registered and compared between treatments. The adult escape rate was calculated as the ratio between the number of adult escaped from the suction tube to the total number of adult males processed. Three replicates per each radiation treatment and exposure time were performed.

### Experiment 4: Effect of radiation on adult male quality

The adult survival and mating capacity of the male irradiated at different doses (0, 35, 60 and 80 Gy) were investigated in order to link these quality parameters with the male flight capacity measured by means of the quality control devices described above.

Fertility of adult males irradiated at the different doses was evaluated by testing the hatching rate of eggs laid by 50 virgin females caged with 50 males for a week. Fertility of three samples of about 1000 eggs per treatment was investigated with females at the first gonotrophic cycle.

Longevity of males was recorded by counting and removing dead adults daily from caged samples of 300 males from each radiation treatment. Adult males were provided with constant access to 10% sucrose solution.

Mating capacity of males from each radiation treatment was investigated by determining the number of females a single male could successfully inseminate over five days. A single virgin male aged 24 h was exposed to 10 receptive virgin females in a plastic cage (20 x 20 x 20 cm) for five days with continuous access to 10% sucrose solution. After exposure, each female was dissected and the number of mated females and filled spermathecal capsules were recorded. Seven replicates per radiation treatment were performed. Females were considered inseminated when they presented at least one spermatheca filled with sperm. A full insemination status was declared when the presence of sperm was detected in at least two spermathecae.

### Statistical analysis

All statistical analyses were performed using IBM SPSS Statistics 23 (IBM Corporation, Armonk, NY) with an alpha level of 0.05. Logarithmic transformation of count data and angular transformation of proportions were performed to meet assumptions of normality (Kolmogorov-Smirnov) and homogeneity of variance (Levene’s Test) prior to analysis. All statistical analyses were based on transformed data, and back-transformed values are presented in the text and in the figures to aid interpretability.

Adult emergence rate, escape rate and time, fertility rate, insemination rate were analyzed by means of general linear model with Tukey's post-hoc pairwise comparisons for means separation considering the effect and interactions of pupal settings, the height of the tubes, the radiation doses and the observation times as the main factors.

Kaplan-Meier survival analysis was used to determine the males’ survival curves at five and thirty days for all treatments groups. Data sets were compared using the Mantel-Cox log-rank test.

We then tried to test the results of flight ability and resistance to stress to predict survival and insemination rates. Experiment 4 was thus used as a reference test. A binomial linear mixed effect model was used to analyze the full insemination rate. The full insemination rate was thus used as the response variable and we built various models with the different escape rates used as fix effects to predict this response. The replications were considered as a random effect. The best model was selected on the basis of the lowest corrected Akaike information criterion (AICc) [21, 22]. The R2 (coefficient of determination) was used to describe the proportion of variance explained by the models [23].

The same procedure was applied to the insemination rate as response variable. Gaussian linear mixed effects models were used for the number of spermatheca inseminated per female, the survival time at day 5 and the survival time at day 30.

## Results

### Experiment 1: Flight organ validation tests

The adult emergence rate obtained with pupae maintained inside the basal plate wells with and without the restriction of the tubes (0.984, 95% CIs 0.945–0.999 and 0.952, 95% CIs 0.885–0.998, respectively) were similar (F_2,17_ = 0.741; p > 0.05) to that obtained from adult emerged from pupae grouped inside Petri dishes (0.986, 95% CIs 0.948–0.999).

**Fig 2:**
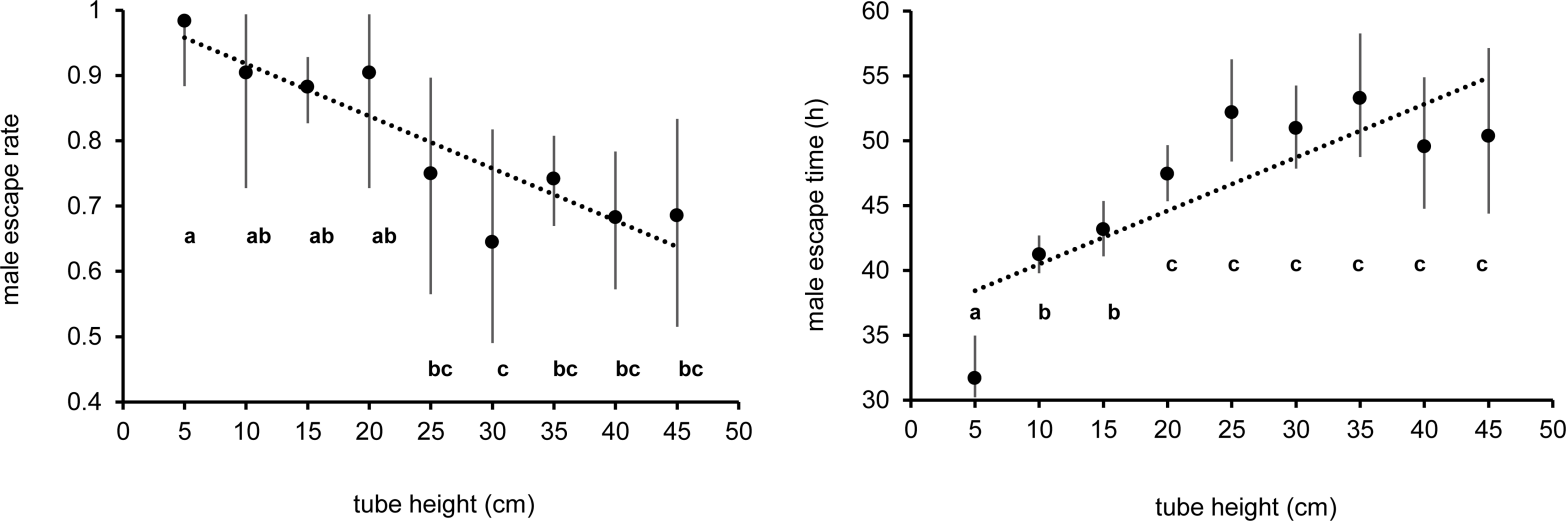
Mean male escape rate (left) and time (right) from flight organs with different tubes heights. Mean represents back-transformed data and bars indicate 95% CIs. Different letters represent statistically significant differences among means at p < 0.05 level (GLM with Tukey's HSD).

The escape rate (F_8,36_ = 7.16; p < 0.001) and the escape time (F_8,36_ = 32.12; p < 0.001) of males from the flight organ differed with the height of the tubes employed (Fig 2). Linear regression analysis of the escape rate and time on the height of the flight organs tubes demonstrated significant relationships (R^2^ = 0.48, F_1,43_ = 39.6; p < 0.001 and R^2^ = 0.57, F_1,43_ = 57.3; p < 0.001 respectively). For every 1-cm increase in the mean height of the tubes, we observed a decrease of 0.7% in the male escape rate (ER = -0.007 cm + 0.973, Fig 2 left) and an increase of 0.42 h in the male escape time (ET = 0.412 cm + 34.36, Fig 2 right). The escape rates recorded from tubes equal to or less than 20 cm were not statistically different (F_3,16_ = 1.68; p > 0.05). Similarly, tubes of a height between 25 and 45 cm did not induce significant differences in the percentages of escape of males (F_4,20_ = 0.69; p > 0.05).

Adults placed in tubes of 5 cm height showed the lowest escape time from adult emergence (mean 31.7 h, 95% CIs 30.3–34.9). The male escape time from tubes of 10 and 15 cm were not statistically different with an overall escape time equal to 42.2 h (95% Cis 40.9–43.4). When males were positioned in tubes equal to or higher than 20 cm they spent an average of 50.6 h to escape (95% Cis 49.2–52.1) without statistical differences between the different tube heights.

The differences observed in the adult male escape rate and time suggested the use of 20 cm as the minimum discriminant height to detect statistical differences in adult male flight ability.

### Experiment 2: Effect of radiation, tube height and time on adult male flight ability

**Fig 3:**
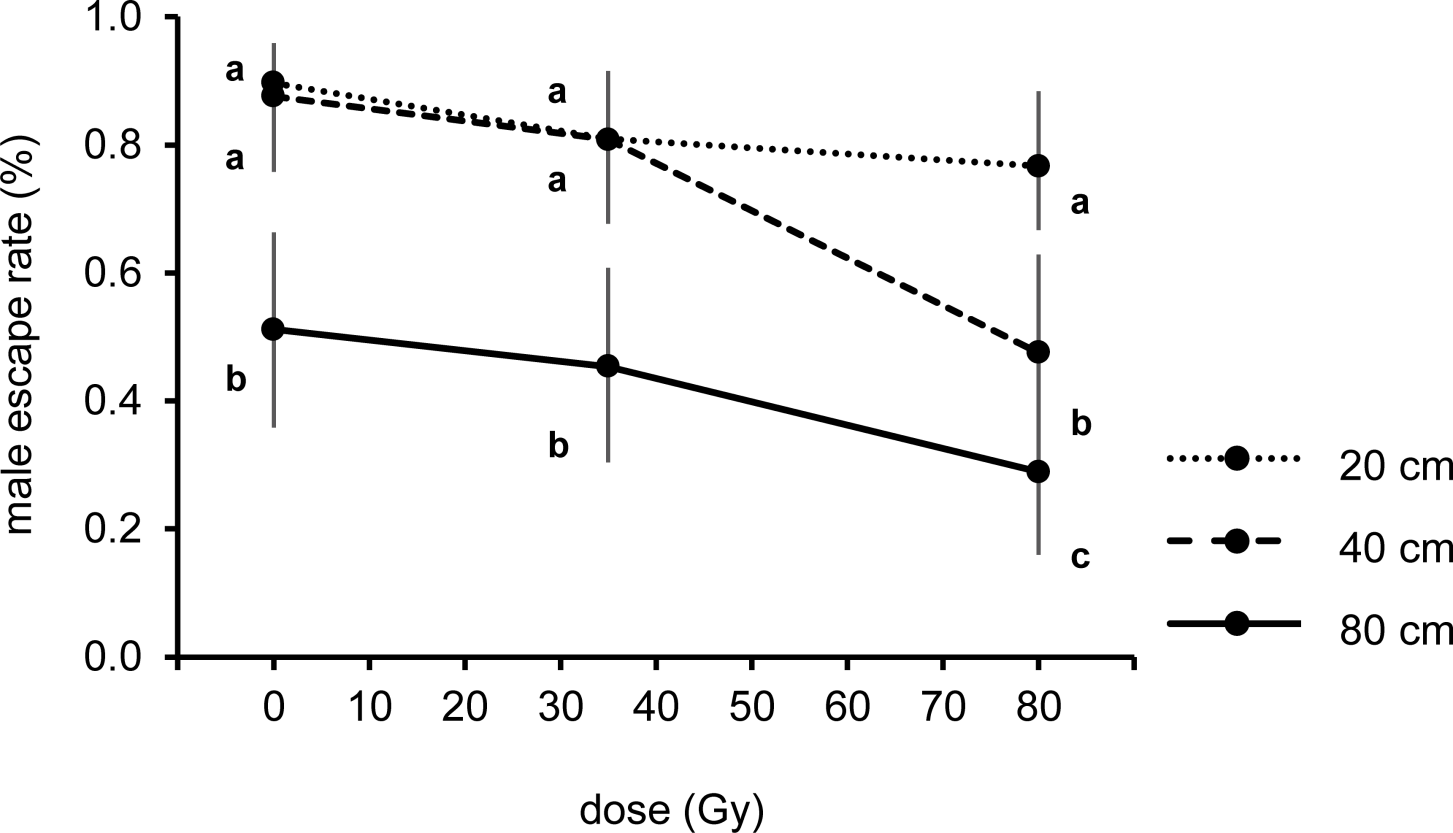
Mean escape rate of irradiated (35 and 80 Gy) and unirradiated males from flight organs with different tubes heights at 48 h from pupal introduction. Mean represents back-transformed data and bars indicate 95% CIs. Different letters represent statistically significant differences of means at p < 0.05 level (GLM with Tukey's HSD) between treatments at the same tube height or dose groups.

In the preliminary test, the height of the tubes (F_2,18_ = 58.79; p < 0.001) and the radiation doses administered to the male at pupal stage (F_2,18_ = 22.27; p < 0.001) significantly influenced the adult escape rate at 48 h from the introduction of the pupae (Fig 3). A statistical interaction between these factors (F_4,18_ = 3.11; p < 0.05) indicated that the male escape rate at different heights depends on the radiation dose received (Fig 3).

Irradiation of pupae at the three tested doses did not affect the capacity of the adult males to escape from tubes of 20 cm (F_2,18_ = 2.03; p > 0.05). The adult escape rate from tubes of 40 and 80 cm showed statistical differences according to the radiation dose received (F_2,18_ = 20.50; p < 0.001 and F_2,18_ = 5.96; p < 0.05, respectively). Males irradiated at 80 Gy were less able of escaping from tubes of 40 and 80 cm height in comparison with both untreated (MD = 0.401; p < 0.001 and MD = 0.223; p < 0.01) and 35 Gy irradiated males (MD = 0.333; p < 0.001 and MD = 0.165; p < 0.05). Tubes of 80 cm height significantly reduced the escape rate at all the doses tested in comparison with the 20 cm tube (0 Gy: MD = 0.385; p < 0.001, 35 Gy: MD = 0.346; p < 0.05, 80 Gy: MD = 0.478; p < 0.001 respectively) and 40 cm height (0 Gy: MD = 0.365; p < 0.001, 35 Gy: MD = 0.354; p < 0.001, 80 Gy: MD = 0.187; p < 0.01 respectively) (Fig 3).

No significant differences were found in the mean escape rate (MD) between untreated and 35 Gy irradiated males at all the tube heights tested (20 cm: MD = 0.097; p > 0.05, 40 cm: MD = 0.068; p > 0.05 and 80 cm; MD = 0.058; p > 0.05) (Fig 3).

**Fig 4:**
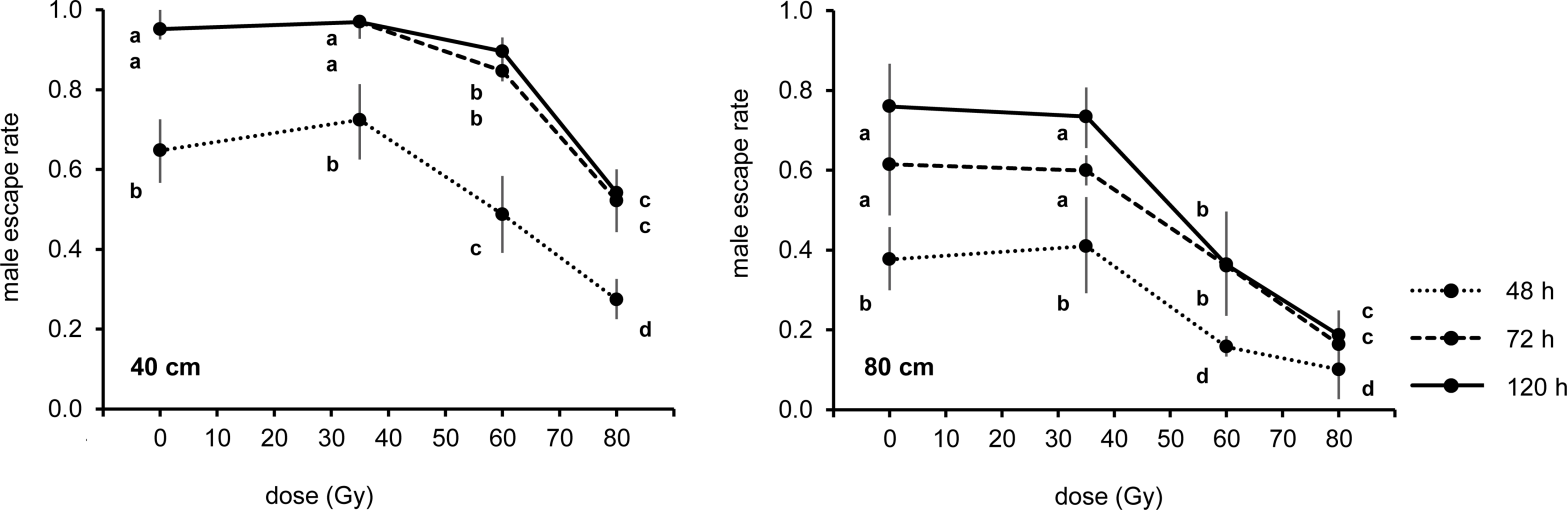
Mean escape rate of irradiated and unirradiated males from flight organs with tubes of 40 cm (left) and 80 cm (right) at 48 h, 72 h and 120 h from pupal introduction. Mean represents back-transformed data and bars indicate 95% CIs. Different letters represent statistically significant differences of means at p < 0.05 level (GLM with Tukey's HSD) between treatments at the same time or dose groups.

The radiation dose, height of the tube and time after introduction all had significant effects on the escape rate of males in the second test (F_3,57_ = 81.31; p < 0.001; F_1,57_ = 235.93; p < 0.001 and F_2,57_ = 64.06; p < 0.001, respectively). A significant interaction was observed between the height of the tube and the radiation dose (F_3,57_ = 3.45; p < 0.05). The escape rates of males from tubes of 40 and 80 cm height was affected by the radiation treatments received at pupal stage at (F_3,57_ = 42.58; p < 0.001 and F_3,57_ = 40.27; p < 0.001) and by the time after introduction (F_2,57_ = 51.10; p < 0.001 and F_2,57_ = 18.97; p < 0.001) (Fig 4).

Similarly to results obtained in the previous test, the dose of 35 Gy did not affect the capacity of males to escape from tubes of 40 and 80 cm height in comparison with untreated males (MD = 0.059; p > 0.05 and MD = 0.004; p > 0.05) for all times after introduction (Fig 4). Males irradiated at the highest dose of 80 Gy showed a lower escape rate from tubes of 40 and 80 cm in comparison with untreated males (MD = 0.483; p < 0.001 and MD = 0.478; p < 0.001) and males irradiated at 35 (MD = 0.542; p < 0.001 and MD = 0.474; p < 0.001) and 60 Gy (MD = 0.333; p < 0.001 and 0.171; p < 0.05). Radiation dose of 60 Gy significantly but less intensely affected the escape rate of males from tubes of 40 and 80 cm height in comparison with untreated (MD = 0.163; p < 0.05 and MD = 0.307; p < 0.001) and 35 Gy irradiated males (MD = 0.209; p < 0.01 and MD = 0.303; p < 0.001) (Fig 4).

The escape rates at 48 h from tubes of 40 and 80 cm were lower compared with the observations recorded at 72 h (MD = 0.361; p < 0.001 and MD = 0.190; p < 0.001) and 120 h (MD = 0.384; p < 0.001 and MD = 0.274; p < 0.001). The escape rates of males observed at 72 h and 120 h were similar in tubes of 40 (MD = 0.023; p > 0.05) and 80 cm height (MD = 0.084; p > 0.05) (Fig 4).

### Experiment 3: Effect of radiation on adult male stress resistance

The escape rate of adult males from the aspirator device was affected by the radiation doses administered at the pupal stage (F_3,23_ = 24.29; p < 0.001) and by the aspiration time (F_2,23_ = 19.54; p < 0.001).

**Fig 5:**
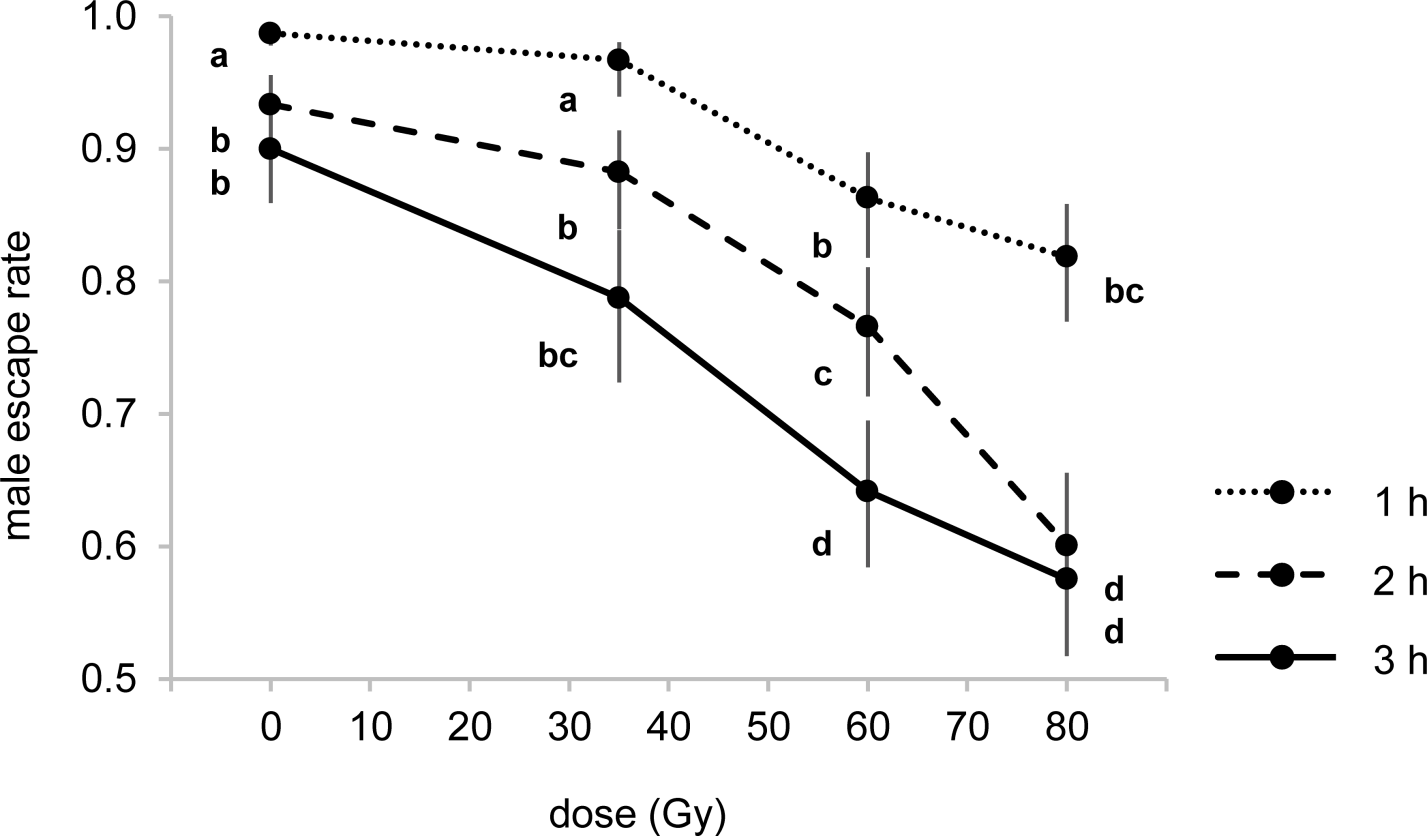
Mean escape rate of irradiated and unirradiated males from the aspirator device after 1h, 2h and 3h of aspiration. Means represent back-transformed data and bars indicate 95% CIs. Different letters represent statistically significant differences of means at p < 0.05 level (GLM with Tukey's HSD) between treatments at the same time or dose groups.

The overall male escape rate was higher after one hour of aspiration than after 2 h (MD: 0.121; p < 0.001) and 3 h (MD: 0.194; p < 0.001) while no statistical differences were observed between 2 h and 3 h (MD: 0.073; p > 0.05). Untreated and 35 Gy irradiated males escaped equally from the aspirator device at all aspiration times tested and with an overall higher rate in comparison with males irradiated at 60 (MD: 0.086; p< 0.001 and MD: 0.029; p < 0.05) and 80 Gy (MD: 0.151; p< 0.001 and 0.073; p< 0.001). Statistical differences were observed between the escape rate of males irradiated at 60 and 80 Gy only after 2 h of aspiration (MD = 0.158; p < 0.05) (Fig 5).

### Experiment 4: Effect of radiation on adult male quality

The fertility of adult males was affected by the radiation dose received at the pupal stage (F_3,8_ = 808.9; p < 0.001). As expected, males irradiated at 35 Gy showed a residual fertility rate (mean 0.020, 95% CIs 0.006–0.046) statistically lower (MD = 0.786; p < 0.001) compared with the values recorded from females mated with untreated males (mean 0.806, 95% CIs 0.688–0.924). No hatching was recorded from eggs laid by females caged with males irradiated at 60 and 80 Gy.

**Fig 6:**
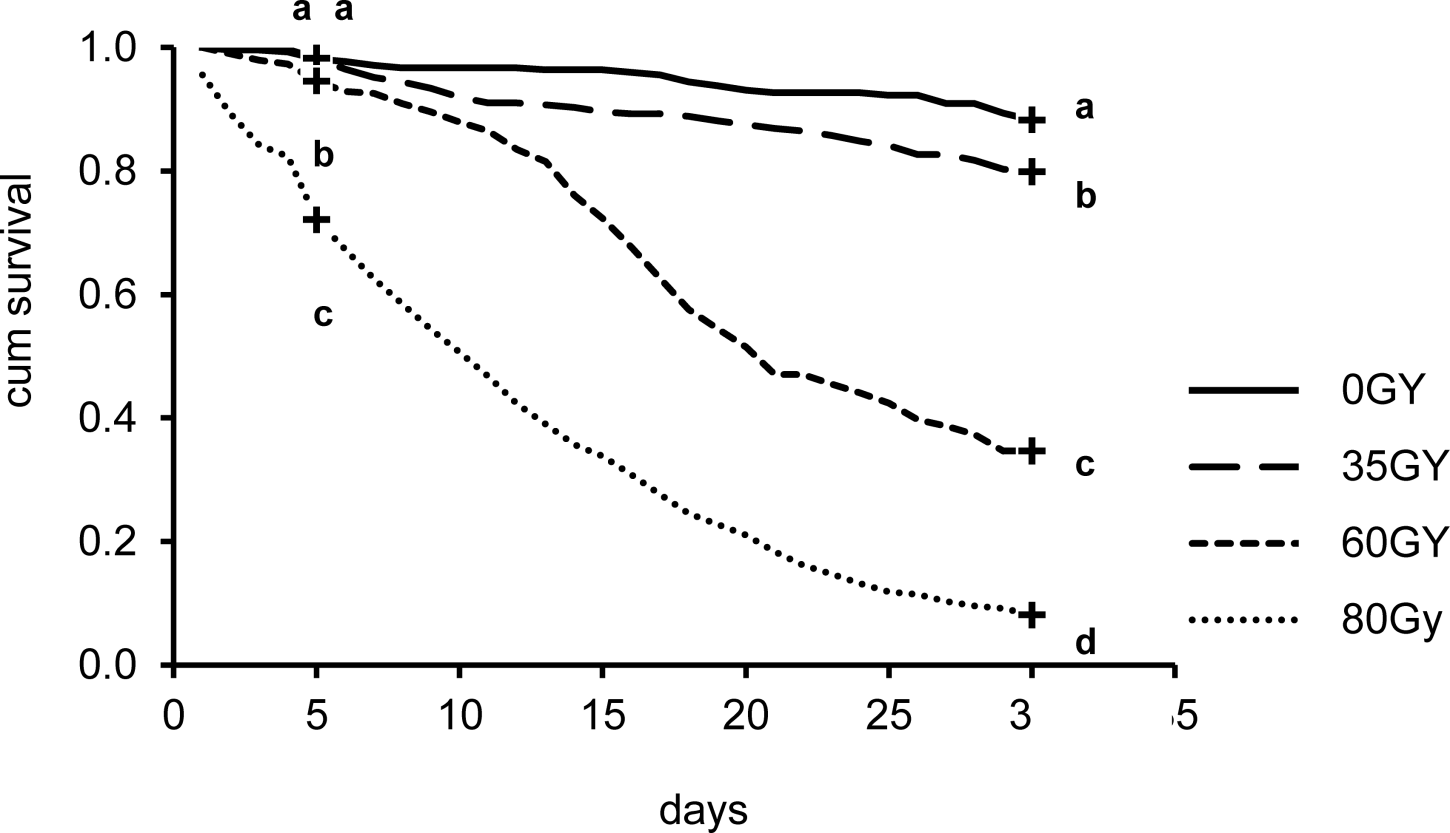
Survival curves of irradiated and unirradiated males. Crosses at day 5 and day 30 indicate censored data at the selected time for survival comparison between treatments. Different letters represent statistically significant differences of survival distributions at p < 0.05 level (Mantel-Cox log-rank test) for the different levels of radiation.

The adult male survival distributions calculated at 5 and at 30 days from the introduction of the pupae in the cages were significantly different between the radiation treatments performed (Log-Rank: chi-square = 171; df = 3; p < 0.001 and Log-Rank: chi-square = 704; df = 3; p < 0.001). While at 30 days survival curves showed statistical differences between all treatments (0.001 < p < 0.01, Fig 6), at day 5 no differences were observed between untreated and 35 Gy irradiated males (Log-Rank: chi-square = 0.01; p > 0.05) while comparisons of all other treatments differed significantly (0.01 < p < 0.05, Fig 6).

**Fig 7:**
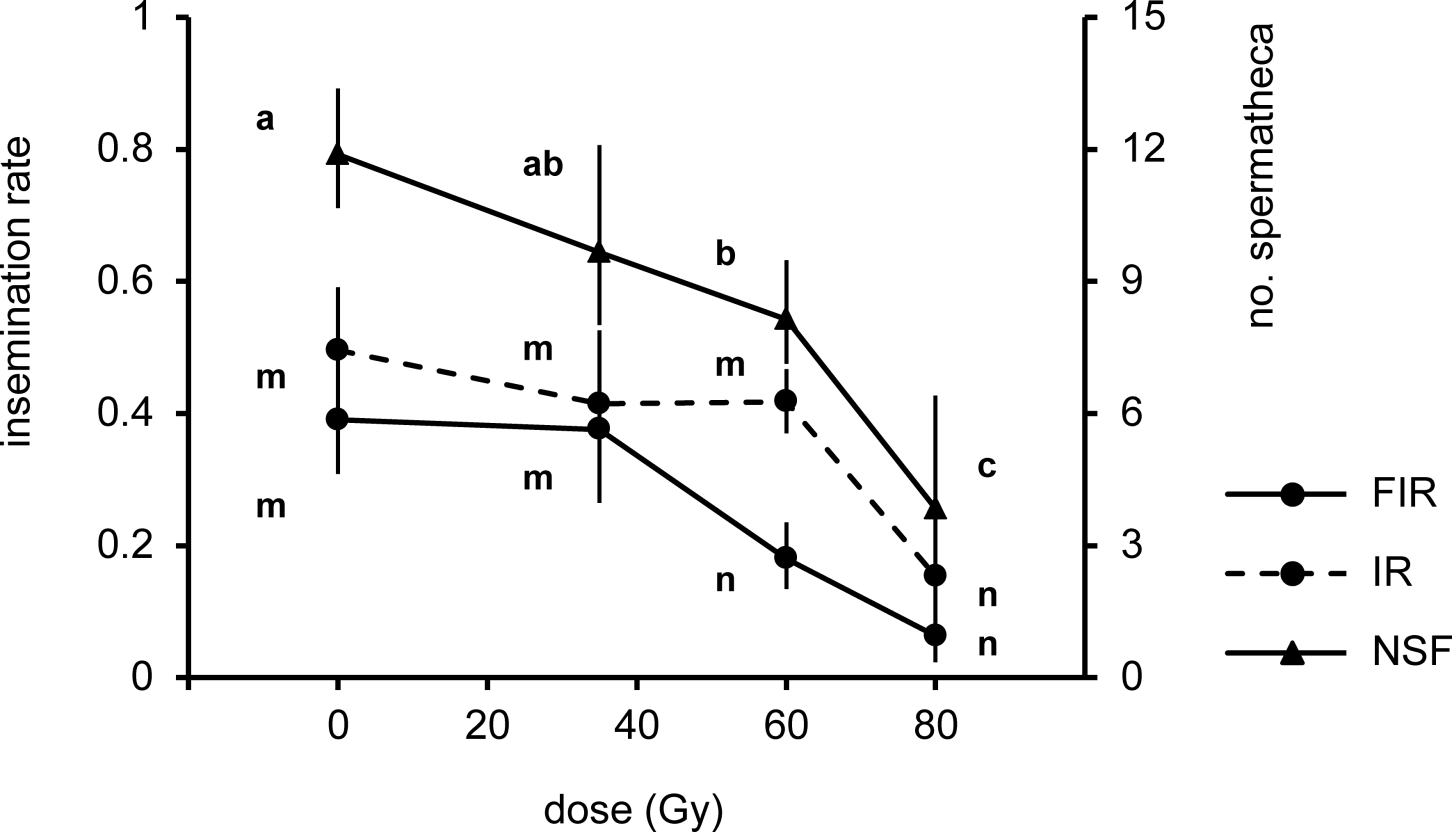
Mean insemination rate (IR; left y-axis), full insemination rate (FIR; left y-axis) and overall number of spermathecae filled (NSF; right y-axis) by a single irradiated or unirradiated male caged with 10 females during a 5 day period. Means represents back-transformed data and bars indicate 95%CIS. Different letters represent statistically significant differences of means at p < 0.05 level (GLM with Tukey's HSD) between treatments per each parameter plotted.

The capacity of males to fill spermathecae (F_3,23_ = 6.653; p < 0.001), inseminate (F_3,23_ = 3.26; p < 0.05) and fully inseminate females (F_3,23_ = 6.696; p < 0.001) was affected by the dose received at the pupal stage. Males irradiated at 35 and 60 Gy were able to inseminate (IR) the same number of females in comparison with untreated males (MD = 0.081; p > 0.05 and MD = 0.078; p > 0.05) but only the former were capable of filling the same overall number of spermathecae (NSF) and fully inseminated (FIR) females at the same rate reported in females mated with untreated males (MD = 1.98; p > 0.05 and MD = 0.02; p > 0.05) (Fig 7). The dose of 80 Gy impair the capacity to fill spermathecae, to inseminate and to fully inseminate females in comparison with untreated males (MD = 7.00; p < 0.001, MD = 0.25; p < 0.05 and MD = 0.28; p < 0.001) (Fig 7).

**Fig 8:**
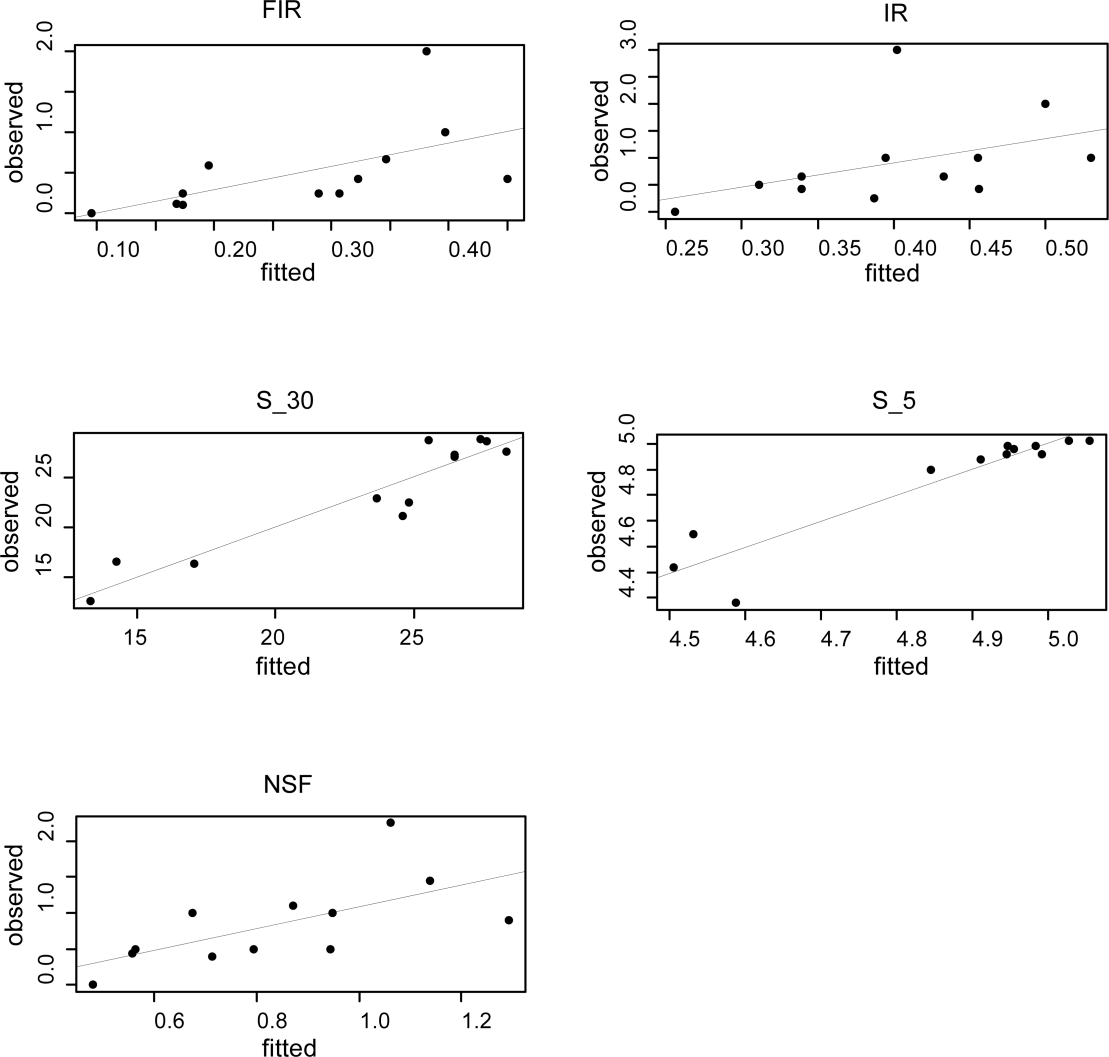
Relationships between observed quality parameters and predicted values using male escape rates. For each panel, the best model highlighted by an asterisk in Table 1 is presented. Mean male survival time at five days (S_5) and 30 days (S_30). Insemination rates (IR), full insemination rate (FIR) and number of spermatheca filled (NSF) by a single male caged with 10 females during a 5 day period.

According to the AICc, the capacity of irradiated males to inseminate (IR) and fully inseminate females (FIR) was best predicted by models employing the male escape rate from flight organ tubes 80 cm high at 120 h from pupal introduction (80_3) as response variable (Tab. 1). These models explained respectively 58% and 76% of total variance observed for IR and FIR respectively (Tab. 1). The low difference in AICc between all the FIR models including escape rate from flight organs of 40cm at 48h show that the latter might still be used to obtained faster estimates [22]. The male escape rate observed with all quality control devices can similarly explain the capacity of irradiated males to transfer sperm to females (AICc: 31.5–34) with the highest predictive ability obtained in the model employing the dataset of adult flight performance after 1h of aspiration (A_1; Tab 1). This model however predicted only 42% of the total variance. The escape rate of irradiated males from flight organ tubes 40 cm high at 72 and 120 h were the best explanatory variables to predict the mean survival rate of irradiated males respectively at 30 and 5 days with models explaining more than 80% of the total variance (Tab 1). The models that best explain the relationships between observed adult male quality parameters and predicted values using male escape rates (*, Tab 1) are depicted in Fig 8.

**Table 1:** Use of the male escape rates from the flight organ and the aspirator devices to predict adult male quality parameters.

	FIR	IR	NSF	S_5	S_30
**40_1**	51.4 (0.47)	55.4 (0.53)	33.6 (0.35)	1.0 (0.74)	65.5 (0.74)
**40_2**	51.8 (0.50)	55.7 (0.49)	33.6 (0.35)	-3.8 (0.86)	57.0 (0.89)*
**40_3**	51.7 (0.50)	55.7 (0.49)	33.5 (0.35)	-5.1 (0.87)*	57.5 (0.88)
**80_1**	50.5 (0.62)	55.7 (0.50)	33.1 (0.36)	7.3 (0.46)	69.4 (0.61)
**80_2**	49.8 (0.55)	55.5 (0.46)	33.8 (0.33)	2.6 (0.68)	63.2 (0.80)
**80_3**	48.9 (0.76)*	55.0 (0.58)*	34.0 (0.36)	5.2 (0.61)	61.4 (0.84)
**A_1**	52.3 (0.46)	55.2 (0.54)	31.5 (0.42)*	7.4 (0.42)	65.6 (0.70)
**A_2**	52.0 (0.53)	55.7 (0.52)	33.2 (0.34)	7.5 (0.48)	67.7 (0.75)
**A_3**	50.1 (0.71)	55.2 (0.61)	32.7 (0.39)	7.8 (0.48)	64.1 (0.82)

The Akaike information criterion (AICc) is presented to compare the ability of the flight parameters used as fixed effects (rows) to predict the quality parameters considered as response variables (columns). The proportion of explained variance is presented in brackets with asterisks identifying response variables producing the best-fit models for the different adult male quality parameters. Escape rate from the flight organ with tubes of 40 cm at 48 h (40_1), 72 h (40_2) and 120 h (40_3) after pupal introduction. Escape rate from the flight organ with tubes of 80 cm at 48 h (80_1), 72 h (80_2) and 120 h (80_3) after pupal introduction. Escape rate from the aspirator device after 1 h (A_1), 2 h (A_2) and 3 h (A_3) of adult aspiration. Mean male survival time at five days (S_5) and 30 days (S_30). Insemination rates (IR), full insemination rate (FIR) and number of spermathecae filled (NSF) by a single male caged with 7 females during a 5 day period.

## Discussion

The capacity of the released sterile males to effectively survive, disperse, compete and inseminate wild females is an essential prerequisite to be evaluated in any AW-IPM programs including a sterile insect release component. Effective emergence and survival rates together with adequate flight and mating capacities need to be regularly monitored and assured throughout all the sterile insect release program. Adequate quality control tests supported by standardized procedures need therefore to be developed to effectively measure these parameters and to identify and correct any inappropriate rearing or handling methods affecting the overall male quality [9].

While variation in fecundity and fertility could indicate detrimental colonization conditions or negative behavioral trait selection in the colonies [7, 14, 24], inconsistent immature size, sex ratio distortion, adult emergence rate and time, survival, flight capacity, mating ability and competitiveness can be related to improper, uncontrolled or changeable immature rearing conditions, handling methods or sterilization procedures [8, 9, 25, 26].

Radiation is the most commonly used method to induce reproductive sterility in insects in SIT operational programs without showing any resistance over more than 60 years of continuous applications [27, 28]. Ionizing radiation has, however, been reported to affect insect survival, mating capacity and competitiveness [4, 29, 30] and the optimal radiation dose and development stage for radiation has to be carefully identified in order to maintain appropriate male quality [9].

In this study, we used radiation doses administered at the pupal stage to induce sterility and measured negative impacts on adult male mosquito flight capacity and stress resistance. Together with the standard dose of 35 Gy, successfully employed in SIT pilot field release trials against *Aedes albopictus* [31], we tested radiation treatment at 60 and 80 Gy which were previously identified to affect male survival and mating capacity [17, 32].

Our study demonstrated the possibility of measuring and comparing the effects of radiation on adult male flight capacity by use of the two quality control devices proposed, the flight organ and the aspirator device.

Because of emergence time uncertainty due to pupal age variability at the time of irradiation and the emergence precocity observed in this species after irradiation [12], we decided to observe our flight organ test at different time from pupal introduction. Results obtained consistently indicate comparable flight capacity and quality parameters between untreated and 35 Gy irradiated males while a negative impact was observed with higher radiation doses at all observation time performed. As expected, radiation dose negatively affected the survival rate and the mating capacity of the adult males irradiated at the pupal stage. The different quality control devices measured a similar radiation dose effect on male flight capacity and all their responses can be successfully employed, with different predictive capacities, to infer the adult male quality parameters such as survival and mating capacity. The best predictions were achieved for survival and full insemination rate and these two factors are very important components of male competitiveness.

The escape rate of males from tubes of 40 cm height shows the highest predictive capacity for adult male survival but a reduced ability to infer the capacity of males to inseminate and transfer sperm.

However, the calculated AIC values for each linear mixed effects model obtained using male escape rate at 40 cm height differed by less than 2 AIC units from all the best-fitting models calculated with the other quality control devices and can therefore be equally employed as effective quality predictive models [22]. The use of flight organ with 40 cm tube height is easier to manage and settle as laboratory quality control tool and the observation of the presence of adults inside small vertical tubes is more accurate and practical.

In our study, males irradiated at 35 Gy showed a residual fertility of about 2% while complete sterility was obtained with doses equal to or higher than 60 Gy. The sterility levels obtained in our tests are in agreement with earlier studies performed on *Ae*. *albopictus* using both gamma and x ray sources at pupal stage [17, 32, 33].

As previously reported, increasing the radiation dose to reach complete male sterility causes a measurable reduction in male competitiveness that affects male mating capacity and the effectiveness of SIT programs [9].

However, a dose of 35 Gy induces damages in the treated males that can be detected by observing the different survival rate of the irradiated males at 30 days compared to the untreated males. As previously observed [33, 34], radiation damage did not alter the quality in the first 5 days (120 h) after irradiation, which represents the maximum age of males tested in our quality control trails. Considering that in SIT programs sterile male releases should presumably be performed at least on a weekly basis, this negative effect on male survival may have limited relevance for the effectiveness of the field application of this technique.

The results obtained in this study confirmed the accuracy of the radiation dose selected after laboratory, semi-field and field trials for the application of the SIT against *Ae*. *albopictus*. Males irradiated with a dose of 35 Gy were capable of effectively dispersing, locating a mating arena and effectively competing for mating and so reducing field density of isolated wild populations [31].

The flight performance and quality parameters of males observed in this study were obtained under the experimental conditions described above and different rearing, handling, transportation and sterilization procedures could alter their relationship or modify the predictive ability of the models described. Improper larval rearing conditions, excessive handling or mechanical processing and sexing, packaging for sterilization, transportation and release are all important factors that can produce a detrimental impact on adult male quality. The escape rate of adult males after a single or cumulative exposure to all these factors at the immature stages will need to be established with the proposed quality control devices to identify the presence of the same relationship between flight capacity and male quality parameters.

It is therefore possible that the survival rate differences observed between irradiated and unirradiated males will become more evident and detectable earlier after the adoption of mechanized or enlarged mass rearing procedures.

The observance of quality control standards along sterile insect release programs can generate essential information on rearing productivity and quality consistency, but do not provide an absolute measure of vigor, male competitiveness or mating capabilities in natural conditions [14]. An appropriate threshold range for quality control tests needs to be established based on the performance of sterile males capable of inducing sterility in the field. However, the flight ability comparison between mass produced sterile males and males collected in the target field area at the pupal stage could provide a preliminary estimation of male quality compatibility prior to the start of release activities.

As demonstrated in this study, the proposed flight organ device can also measure the emergence rate and the sex ratio of the pupae sample under evaluation. These two parameters are also extremely important to assess the quality of the males and the efficiency of the sex separation method in any mosquito sterile insect release programs [9]. The use of the aspirator as a rapid quality control device could be extremely helpful to identify appropriate adult male quality before release with the possibility to cancel or delay expensive release procedures and apply recovery stress techniques as described in other mass produced insect for SIT applications [35].

The aspirator device was used in this study to create a mechanical and desiccation stress to the adult males processed. As previously reported [36], adult desiccation resistance may be a factor that influences distribution, abundance and adult survival capacity of *Aedes* (Stegomyia) species and adult longevity under desiccation conditions was reported to be strongly associated with adult nutrient sources accumulation and energy metabolism [37]. Resistance to desiccation therefore deserve further investigations and the use of the aspirator device could reveal important adult quality indications on the nutritional status of the insect produced.

Simpler than a flight mill, the only available instrument for accurate evaluation of insect flight potential [10, 38], our quality control devices are less expensive and laborious, do not need electronic instrumentation and can process several individuals simultaneously. Moreover, in our devices, mosquitoes are not artificially forced to fly therefore providing the first tools for flight capacity and propensity measurement. However, attempt to conduct comparison between our quality control devices and flight mills would be useful to investigate if different flight capacity measures could also reflect differences in flight potential and adult dispersal ability. Finally, the availability of these quality control instruments tested on *Ae*. *albopictus* radio-sterilized males could be useful to identify and compare the quality performance of different mosquito species and strains with different colonization history, origin or genetic background before their amplification and field use in genetic vector control programs including a sterile insect release component.
